# From Plan To Impact – expediting progress towards targets of the WHO Global Action Plan on Dementia

**DOI:** 10.1002/alz.088171

**Published:** 2025-01-09

**Authors:** Paola Barbarino, Diego Aguilar, Lewis Arthurton, Chris Lynch

**Affiliations:** ^1^ Alzheimer’s Disease International, London United Kingdom; ^2^ Alzheimer's Disease International, London United Kingdom

## Abstract

**Background:**

In 2017, all 194 Member States of the World Health Organization (WHO) unanimously adopted the Global Action Plan on the public health response to dementia 2017‐2025, the primary aim being for countries to implement National Dementia Plans (NDP). However, as outlined in Alzheimer’s Disease International’s (ADI) recent From Plan to Impact report, WHO Member States have been far too slow to act ‐ falling far short of the 146 countries target. In response, ADI launched the #WhatsYourPlan campaign, in collaboration with ADI Members, with the single‐minded aim of galvanising governments to act on their 2017 commitment.

**Method:**

Strategizing and working with ADI Members, official communications were sent to relevant ministries to enquire as to the status of the NDP, and to request a formal meeting. For Ministries where a response was not obtained, subsequent letters were sent to elicit a response. ADI has also, in some instances, sought advice and utilised WHO regional and national offices, and other multilateral bodies and stakeholders, to help follow up with Ministries of Health for those that have been willing to engage.

**Results:**

52 ADI members and thus 52 countries, joined the campaign. In total from the onset of the campaign, 205 official communications were shared with Member States, resulting in a total of 29 high‐level meetings. In total 20 Member States committed to developing an NDP, just over a third of all participating ADI Members and over half of the number of plans already in existence in WHO Member States

**Conclusions:**

Though progress on the GAP has been slow, ADI’s successful #WhatsYourPlan campaign demonstrates that collaborative efforts between governments and stakeholders can make a tangible impact on policy development and implementation of NDPs. The fight for a better world for people living with dementia is ongoing, and it is crucial to utilise all available means to transform this vision into reality. As such, ADI is calling for an urgent extension of the Global Action Plan until 2029 with current progress putting people living with dementia and their caregivers are at a severe disadvantage.

